# Vitamin D receptor regulates proliferation and differentiation of thyroid carcinoma via the E-cadherin-β-catenin complex

**DOI:** 10.1530/JME-21-0167

**Published:** 2022-01-31

**Authors:** Yali Ling, Feng Xu, Xuedi Xia, Dexing Dai, Ruoman Sun, Zhongjian Xie

**Affiliations:** 1 National Clinical Research Center for Metabolic Diseases, Hunan Provincial Key Laboratory of Metabolic Bone Diseases, and Department of Metabolism and Endocrinology, The Second Xiangya Hospital of Central South University, Changsha, Hunan, China

**Keywords:** vitamin D receptor, differentiated thyroid carcinoma, proliferation, differentiation, E-cadherin, β-catenin

## Abstract

Thyroid cancer has the fastest rising incidence among cancers, especially for differentiated thyroid carcinoma (DTC). Although the prognosis of DTC is relatively good, if it changes to anaplastic thyroid carcinoma (ATC), the prognosis will be very poor. The prognosis of DTC is largely depending on the degree of cell differentiation and proliferation. However, whether the vitamin D receptor (VDR) plays a role in regulating the proliferation and the differentiation of DTC cells is unclear. In the present study, we found that VDR was upregulated in DTC tissues compared to the adjacent non-cancerous tissue. Knockdown of VDR increased proliferation and decreased differentiation proliferation in DTC cells* in vitro* as well as DTC cell-derived xenografts* in vivo*. In contrast, overexpression of VDR had an opposite effect. Knockdown of E-cadherin abolished VDR-induced suppression of proliferation and enhancement of differentiation of the DTC cells. Knockdown of β-catenin partially reversed the effect of the VDR knockdown. VDR increases the levels of E-cadherin in the plasma membrane and decreases the levels of β-catenin in the nucleus. VDR binds to E-cadherin and β-catenin in the plasma membrane of the DTC cell. Taken together, VDR inhibits DTC cell proliferation and promotes differentiation via regulation of the E-cadherin/β-catenin complex, potentially representing novel clues for a therapeutic strategy to attenuate thyroid cancer progression.

## Introduction

Thyroid carcinoma is one of the most common endocrine cancers worldwide and its incidence has been rising in the past decades, becoming one of the tumors with the fastest increase (Lim *et al.* 2017). It can be divided into papillary thyroid carcinoma (PTC), follicular thyroid carcinoma (FTC), anaplastic thyroid carcinoma (ATC), and medullary thyroid carcinoma. FTC and PTC are also defined as differentiated thyroid carcinoma (DTC), accounting for 90% of thyroid malignancies (Ito *et al.* 2013).

Although most DTC has an excellent prognosis with 80–95% 10-year survival rates but a high risk for long-term harm, the prognosis significantly worsens when the tumor grows, and the degree of tumor differentiation decreases (Fagin & Wells 2016, Filetti *et al.* 2019). Furthermore, ATC accounts for 1% of all thyroid cancers with a poor survival (Ain 1998, Fagin & Wells 2016). For these reasons, it is important to understand the characteristics of the tumor at the early stage and to better prevent the progression of the tumor.

The vitamin D receptor (VDR), a well-known member of the nuclear receptor superfamily, mediates the action of 1,25-dihydroxyvitamin D_3_ (1,25(OH)₂D_3_). VDR is widely expressed in multiple kinds of cells, playing an important role in cell differentiation and proliferation as well as apoptosis (Mehta & Mehta 2002). More recently, growing evidence has demonstrated that VDR protects against tumor progression of a number of common cancers such as prostate cancer, gastric cancer, and breast cancer (Thorne & Campbell 2008). VDR knockout mice displayed an increased incidence of carcinogen-induced tumors in breast, skin, and lymphoid tissue (Zinser *et al.* 2005, Ooi *et al.* 2010). Furthermore, VDR polymorphism has been associated with the incidence of cancers (Giovannucci *et al.* 2006, Gandini *et al.* 2014). It has been shown that VDR is strongly expressed in DTC tissues compared with adjacent normal thyroid tissues, but is lower or absent in human ATC tissues (Clinckspoor *et al.* 2012, Izkhakov *et al.* 2016). VDR has been shown to inhibit the growth of several types of tumors by directly or indirectly regulating cell cycling and proliferation, differentiation, and apoptosis. However, the significance of the elevated VDR levels in DTC and the role of VDR in regulating DTC proliferation and differentiation remains unknown.

In addition to the role of VDR in mediating 1,25(OH)_2_D_3_-regulated cell proliferation and differentiation, VDR regulates cell differentiation and proliferation in a ligand-independent and non-genomic fashion in some cell types. It has been reported that mice with an overexpression of the ligand-binding site of VDR in the epidermis restored normal hair cycling in VDR knockout mice (Skorija *et al.* 2005), suggesting that the role of VDR in epidermal hair regulation is independent of its ligand. VDR interacts with β-catenin to regulate the transcription of target genes, thus participating in the regulation of keratinocyte stem cell function (Cianferotti *et al.* 2007).

E-cadherin is an adhesion molecule and plays an important role in regulating different cellular processes (Dongre & Weinberg 2019). β-catenin is bound mostly to the cytoplasmic domain of E-cadherin at the adherens junction in normal epithelial cells. Previous studies have revealed an association of the reduction of E-cadherin with stronger nuclear staining for β-catenin in advanced malignancies (Tafrihi & Nakhaei Sistani 2017). Besides, E-cadherin is expressed in normal thyroid and PTC but decreased and even lost in ATC (Brabant *et al.* 1993). In thyroid cancer cells, the level of β-catenin in the plasma membrane decreases in undifferentiated or aggressive thyroid cancer cells compared to well-differentiated thyroid cancer cells (Garcia-Rostan *et al.* 2001). However, the role of E-cadherin-β-catenin signaling in DTC is still not well understood.

The present study sought to determine whether VDR regulates DTC cell proliferation and differentiation via E-cadherin and β-catenin.

## Materials and methods

### Cell culture

Human thyroid cell carcinoma K1 cell line and WRO cell line derived from metastatic tissue of a female patient with PTC and FTC were obtained from the European Collection of Authenticated Cell Culture (ECACC) and American Type Culture Collection, respectively. Human normal thyroid cell lines (Nthy-ori-3-1) were purchased from Cell Bio (Shanghai, China). The cell lines were validated using short tandem repeat (STR) analysis, and they confirmed that there was no mycoplasma contamination in the cells. Cells were cultured in Dulbecco’s modified Eagle medium (DMEM), nutrient mixture F-12 (1:1, by volume) with 10% fetal bovine serum (FBS, Invitrogen), 100 units/mL penicillin, and 100 µg/mL streptomycin (Solarbio, Beijing, China).

### Chemicals and antibodies

The bromodeoxyuridine (BrdU) cell proliferation kit was purchased from Millipore. The short interfering RNAs (siRNA) for E-cadherin and β-catenin siRNA were purchased from RiboBio Company Limited (Guangzhou, China) and transfection reagents for siRNAs were purchased from Qiagen Biotechnology. The adenovirus vector was purchased from Vigene Biosciences (Shangdong, China); Trizol (Ambion) was used for RNA extraction. The cDNA Archive Kit and SYBR Green Master Kit were purchased from Takara Biotechnology. The Mem-PERTM Plus Membrane Protein Extraction Kit was purchased from ThermoFisher Scientific (No. 89842). The NE-PER™ nuclear and cytoplasmic extraction reagents were purchased from ThermoFisher Scientific (No. 78833). The BCA protein assay kit was purchased from Beyotime (Shanghai, China). The TUNEL assay kit was purchased from Roche (Cat No. 11684817910). The protein G plus/protein A agarose suspension was purchased from Merck Millipore. The antibodies used in the immunohistochemical and Western analysis include antibodies against thyroid peroxidase (TPO, Cat#ab109383, Abcam), VDR (Cat No. 12550, Cell Signaling Technology), thyroid-stimulating hormone receptor (TSHR, Cat No. sc-13936), E-cadherin (Cat No. sc-7870), and β-catenin (Cat No. sc-7199) antibodies were purchased from Santa Cruz Biotechnology. The GAPDH (Cat No. 60004-1-Ig), horseradish peroxidase-conjugated affinipuregoat anti-rabbit IgG (Cat No. SA00001-2), and horseradish peroxidase-conjugated affinipuregoat anti-mouse IgG (Cat No. SA00001-1) were purchased from Proteintech (Rosemont, IL, USA).

### Histology and immunohistochemistry

Thyroid tissue specimens were obtained from 20 patients with PTC undergoing thyroid surgery at the Second Xiangya Hospital of Central South University in China. The experiment concerning human specimens was approved by the ethics committee in the Second Xiangya Hospital. Histology and immunohistochemical staining were performed as described previously (Wu *et al.* 2018). Briefly, 4-μm-thick formalin-fixed paraffin-embedded tissue sections were prepared and hematoxylin and eosin (H&E) or with a VDR antibody (1:200 dilution), A EliVisionTM Super Immunohistochemistry kits (Maixin KIT-9921, Maixin, Fuzhou, China) and DAB (Maixin, Fuzhou, China) chromogen were used for immunostaining. The proportion of cells showing VDR immunoreactivity in the nucleus and/or cytoplasm was evaluated independently by two investigators. The thyroid tissue specimens were not gathered for the present study. The sections were coded, and no identifiers were associated with the specimens and the patient consent statement did not apply. The experiment regarding human specimens was approved by the ethics committee in the Second Xiangya Hospital.

### Immunofluorescent staining

Cells were fixed with 4% paraformaldehyde. After a brief wash with PBS, cells were permeabilized with 0.1% Triton X-100 in PBS and blocked with 5% BSA for 1 h at 37°C. Coverslips were incubated with a primary anti-VDR mouse antibody and a primary anti-E-cadherin or anti-β-catenin rabbit antibody at 4°C overnight followed by 1-h incubation with fluorescence conjugated secondary antibodies at room temperature (RT). Images were obtained with the fluorescence microscope (AXIO Vert A1).

### Cell transfection, adenoviral infection, and shRNA lentiviral infection

For cell transfection, K1 cells and WRO cells cultured in the DMEM-F12 medium at 70–80% confluency were transfected with human E-cadherin siRNA, human β-catenin siRNA, or negative control using HiperFect transfection reagent (Qiagen) according to the manufacturer’s protocols. After 24–48 h, cells were harvested for further experiments. For adenoviral infection, cells were infected with an adenovirus expression vector containing VDR cDNA (NM_001017535) (VDR-adv) or negative control adenovirus (control-adv). For the shRNA lentiviral infection, cells were transduced with lentiviruses expressing VDR shRNA and control shRNA. The lentiviral vector GV344 was purchased from Genechem (Genechem, Shanghai, China). The sequence number of shRNA is shown in Table 1. After 24 h at 37°C, cells were transferred to growth medium, and stable cell lines were selected by using puromycin for 5–7 days. Individual control and VDR shRNA-expressing clones were isolated by limited dilution in the presence of puromycin. The knockdown or overexpression efficiency was analyzed by immunoblotting for respective proteins.

**Table 1:** Sequences of VDR shRNA.

VDR	LV-VDR-RNAi (70776–1)
VDR-RNAi (70776–1)-a	5’-ATCTCATTGCCAAACACTTCG-3’
VDR-RNAi (70776–1)-b	5’-ATCTCATTGCCAAACACTTCG-3’
Control-shRNA	TTCTCCGAACGTGTCACGT

### BrdU cell proliferation assay

The proliferation of K1 cells and WRO cells was determined by BrdU cell proliferation assay using a BrdU cell proliferation assay kit according to the manufacturer’s instructions (Millipore). Briefly, cells were plated in 96-well plates at a density of 1 × 10^4^cells/well were cultured for 2 days. Cells were incubated in the serum-free medium supplemented without FBS for a further 12 h, the proliferation assay was performed 12 h following the addition of BrdU reagent at a concentration of 10 ng/mL. Briefly, cells were incubated with anti-BrdU antibody for 1 h and then with peroxidase-conjugated secondary antibodies for 30 min. The reaction product was color developed by incubation with a peroxidase substrate. The absorbance values measured at 450 nm wavelength represented the rate of DNA synthesis and corresponded to the number of proliferating cells.

### Colony formation assays

For colony formation assay, 1 × 10^3^cells were cultured in six-well plates with the medium at 37°C in 5% CO_2_ and colony formation was assessed after 2 weeks. Cells were fixed with 4% paraformaldehyde for 20 min and stained with 0.1% crystal violet for 20 min at the end of the experiment. The visible colonies were counted and imaged.

### Apoptosis by TUNEL assay

Cell apoptosis was evaluated by TUNEL staining on tissue sections using the TUNEL assay kit following the manufacturer’s instructions. Briefly, sections were exposed to 5 μg/mL of proteinase K for 25 min at 37°C and then treated with 0.2% Triton X-100 solution for 20 min at room temperature. The sections were washed extensively and exposed to PBS (pH 7.4). According to the number of sections and tissue size, an appropriate amount of reagent 1 (TdT) and reagent 2 (dUTP) of the TUNEL kit were mixed at 1:9 and added to the covered tissue. The sections were incubated at 37°C for 2 h in a humidified chamber. Each slide was then washed with PBS (pH 7.4) for 5 min and repeated three times. Then DAPI staining solution was added and incubated for 10 min at room temperature in a dark room. The sections after sealing were observed using a fluorescence microscope and the images were collected.

### Gene expression using a quantitative real-time polymerase chain reaction

Total RNA was extracted from K1 cells and WRO cells using Trizol reagent (Invitrogen) according to the manufacturer’s procedure. Total RNA was reversely transcribed into cDNA by the Takara RT-PCR kit. Real-time PCR assays were carried out using the SYBR® Premix DimerEraserTMKit and specific primers. Quantitative real-time PCR (qRT-PCR) was performed according to the manufacturer’s instructions as previously described (Li *et al.* 2020). Data were analyzed by the instrument’s software and were normalized for GAPDH gene levels by the ΔΔCt method. The primer sequences are provided in Table 2.

**Table 2:** Primers used in the present study for qRT-PCR assay.

VDR	Forward: 5’-ATAAGACCTACGACCCCACCTA-3’
	Reverse: 5’-GGACGAGTCCATCATGTCTGAA-3’
TSHR	Forward: 5’-AGCCACTGCTGTGCTTTTAAG-3’
	Reverse: 5’-TCCAAAACCAATGATCTCATCC-3’
TPO	Forward: 5’-ACCTCGACGGTGATTTGCA-3’
	Reverse: 5’-CCGCCTGTCTCCGAGATG-3’
NIS	Forward: 5’-TGCCCTCTCTGAGCCTCAAT-3’
	Reverse: 5’-CCTGATCACAGCTGTCACTGTCT-3’
GAPDH	Forward: 5’- TGATGACATCAAGGTGGTGAAG-3’
	Reverse: 5’-TCCTTGGAGGCCATGTGGGCCAT-3’

### Cell fractionation and membrane protein and nuclear protein extraction

Total cell lysates were obtained by extraction in RIPA buffer (50 mM HEPES, pH 7.4, 1% Triton X-100, 0.1% SDS, 150 mM NaCl, 1 mM EDTA) containing a protease inhibitor cocktail (Roche) as well as 1 mM PMSF. Cell membranes were separated by Mem-PERTM Plus Membrane Protein Extraction kit (Thermo Scientific, No. 89842) according to the manufacturer’s procedure.

Cell nuclear protein extraction was separated by NE-PER Nuclear and Cytoplasmic Extraction Reagents Kit (Thermo Scientific, No. 78833) according to the manufacturer’s procedure. Protein concentration was measured by the BCA Protein Assay kit (Beyotime).

### Co-immunoprecipitation and Western blot analysis

Immunoprecipitation was performed using membrane protein extracts. 100 µg of lysate protein of cell extracts were incubated with 2 μg of anti-VDR (Cat No. 12550, Cell Signaling Technology) antibody or Rabbit IgG (A7016, Beyotime) overnight at 4°C with rotation. Following overnight incubation, the lysate protein/antibody or lysate protein/rabbit IgG mixture was then incubated for 2 h with rotation at 4°C with 20 μL of protein G plus/protein A agarose beads. After several washes with PBS, 5 μL of SDS-PAGE sample loading buffer (P0015, Beyotime) was added to the beads. Samples were heated at 99°C for 5 min and then analyzed by Western blot analysis. Western blot analysis was performed using the whole-cell extracts, subcellular fractions, or washed lysate–antibody–agarose beads mixture. Forty micrograms of protein samples were separated on 8% or 12% SDS-PAGE gel and electro-blotted onto PVDF membranes. The membranes were incubated overnight at 4^o^C with antibodies against TSHR (1:1000), TPO (1:1000), VDR (1:1000), E-cadherin (1:1000), β-catenin (1:1000), and GAPDH (1:2000) for 1 h. After incubation with the primary antibody and a series of washes, the membranes were incubated for 1 h with the appropriate anti-IgG secondary antibody conjugated to horseradish peroxidase in the blocking buffer. Following a second series of washes, bound antibody complexes were visualized using the Super signal ULTRA chemiluminescent kit (Millipore). The quantification of Western blots was performed using ImageJ software.

### Animal studies (xenograft assay)

Six-week-old female severe combined immunodeficient (SCID) mice were purchased from Hunan Slac Laboratory Animal Company and were acclimated for 1 week in specific pathogen-free conditions. To assess the tumorigenicity of cells, the tumor-bearing mice model was conducted as described (Li *et al.* 2020). A total number of 5 × 10^6^ cells were trypsinized and resuspended in PBS and then injected subcutaneously into the left or right rear flank of six SCID mice. There were six mice in each group. VDR shRNA cells or control shRNA cells were injected into each flank of the mouse in group 1, VDR-adv or control-adv were injected in each flank of the mouse in group 2. Tumor dimensions were measured by an external caliper every 7 days for a total of 35 days (five rounds), and their volumes were then calculated based on the modified ellipsoidal formula: Tumor volume = ((length × width^2^ × π)/6) (Tomayko & Reynolds 1989). Mice were given an intraperitoneal injection of BrdU at a dosage of 10 mg/kg 2 h before euthanization at 5 weeks post-injection. The tumor tissues were resected, photographed, weighed, and flash-frozen in liquid nitrogen and tainted with histology and immunohistochemistry to determine the expression levels of VDR, proliferating cell nuclear anitgen (PCNA), Ki67, BrdU, TPO, and TSHR. Slides were viewed and photographed under a light microscope (Olympus DP72) as described previously (Li *et al.* 2020). Animal handling and experimental procedures complied with guidelines established by the Animal Care and Use Committee of The Second Xiangya Hospital, Central South University. The tumor burden did not exceed the recommended dimensions and animals were sacrificed by cervical dislocation.

### Statistical methods

GraphPad Prism v9.0 software (GraphPad, Inc.) was used for statistics. Data are presented as the mean ± s.d. Statistical comparisons were performed using the independent sample *t*-test for two groups and one-way or two-way ANOVA followed by a Bonferroni’s* post hoc* test for multiple groups. A *P* value < 0.05 was considered statistically significant.

## Results

### The expression of VDR in DTC tissue was higher than that of the adjacent non-cancerous thyroid tissue

**Figure 1:**
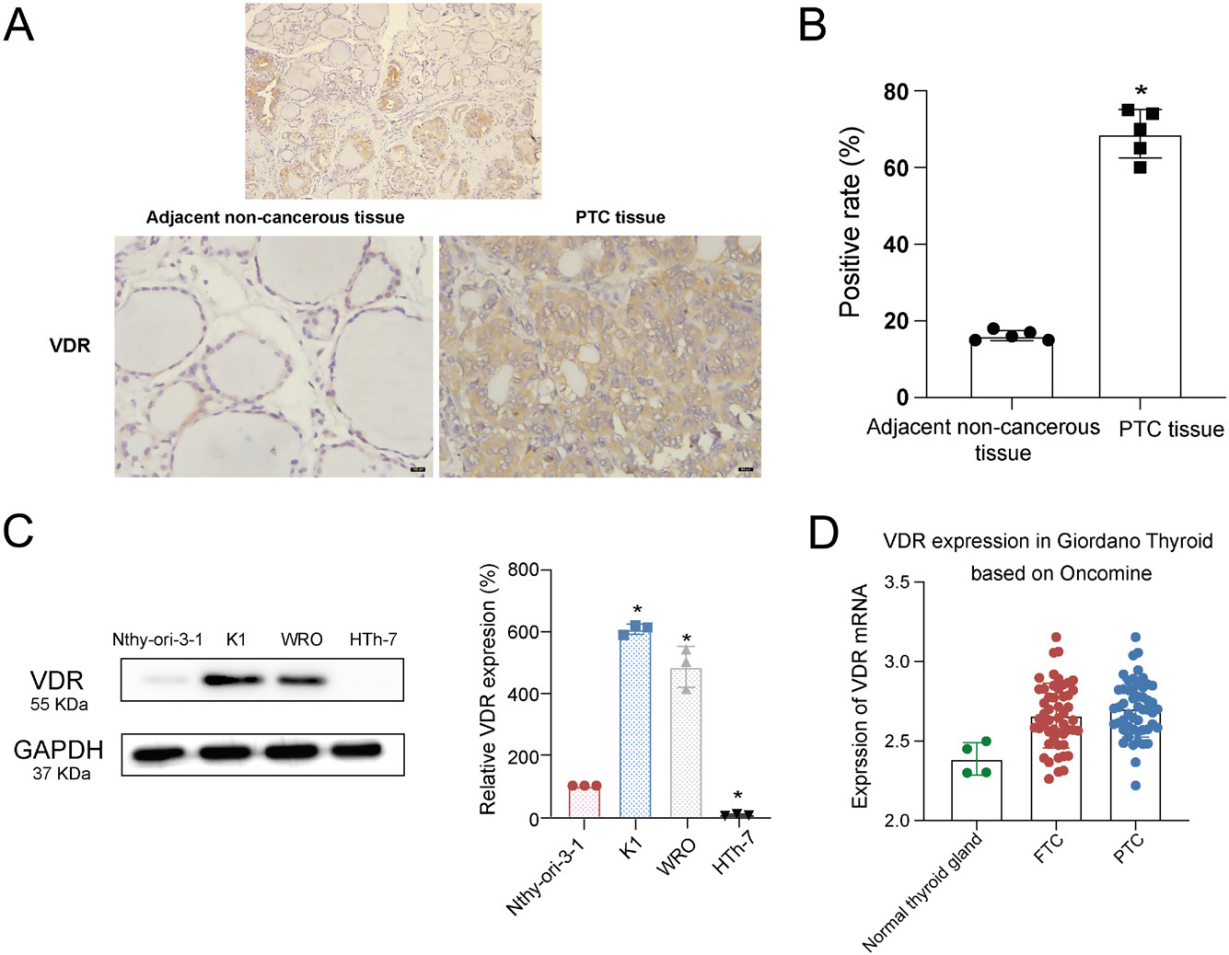
The expression level of VDR in the DTC tissues and cells. (A) Representative IHC staining for VDR in the DTC tissue (*n* = 20) and adjacent non-cancerous tissue (*n* = 20) (The arrow indicates positive staining cells staining brown, magnification, left 100× and right 400×). (B) shows quantification of the cellular localization of VDR in the DTC tissue and adjacent non-cancerous tissue. Positive cells were determined in five randomly selected fields at 400× magnification. Positive cells were counted, and the percentage of positive cells was calculated. (C) The expression of VDR in the normal thyroid cell line (Nthy-ori-3-1), PTC cell line (K1) and FTC cell line (WRO) by Western blotting. Densitometric quantification analysis results were presented in the right panel. The data were presented as densitometric ratios normalized to GAPDH. (D) VDR mRNA expression comparison analysis between the normal thyroid gland and PTC & FTC based on the Oncomine database. Each assay was performed in triplicate. Image J was used for WB quantification. Each assay was performed in triplicate (*n* = 3). Statistical values were analyzed by independent samples *t*-test or ANOVA analysis (*P  < 0.05 vs normal thyroid cells/tissues). A full color version of this figure is available at https://doi.org/10.1530/JME-21-0167.

To determine the expression level of VDR in DTC tissue, we examined the immunostaining level of VDR in 20 paired PTC and adjacent non-cancerous tissues using immunohistochemistry. The results showed that VDR expression was increased significantly in the PTC tissue compared with paired adjacent non-cancerous thyroid tissue, and VDR staining was mainly localized in most cytoplasm and plasma membrane of PTC, whereas VDR staining was localized in the nucleus of paired adjacent non-cancerous thyroid tissue (Fig. 1A). Moreover, we examined the expression of VDR in DTC cell lines (K1, WRO) and human normal thyroid cell lines (Nthy-ori-3-1). The results showed that the expression of VDR was higher in both K1 and WRO cell lines compared with Nthy-ori-3-1 cells (determined by Western blotting). Meanwhile, the K1 cell lines had higher VDR protein expression levels when compared with WRO cell lines (Fig. 1C). Consistently, a higher protein level of VDR was observed in highly differentiated thyroid cancer cell lines (K1 cell). These results were further confirmed through Oncomine datasets analysis by comparing VDR mRNA expression between normal and PTC as well as FTC populations. The upregulated expression of VDR in differentiation thyroid cancer was verified by gene expression analysis (Fig. 1D). We also examined the immunostaining level of VDR in ATC tissues using immunohistochemistry and the results showed that VDR expression was much lower in the ATC tissue compared with DTC tissue (Supplementary Fig. 1, see section on supplementary materials given at the end of this article). Taken together, the expression of VDR was upregulated in DTC tissues and cells.

### VDR suppressed the proliferation and promoted the differentiation of DTC cells *in vitro*

**Figure 2:**
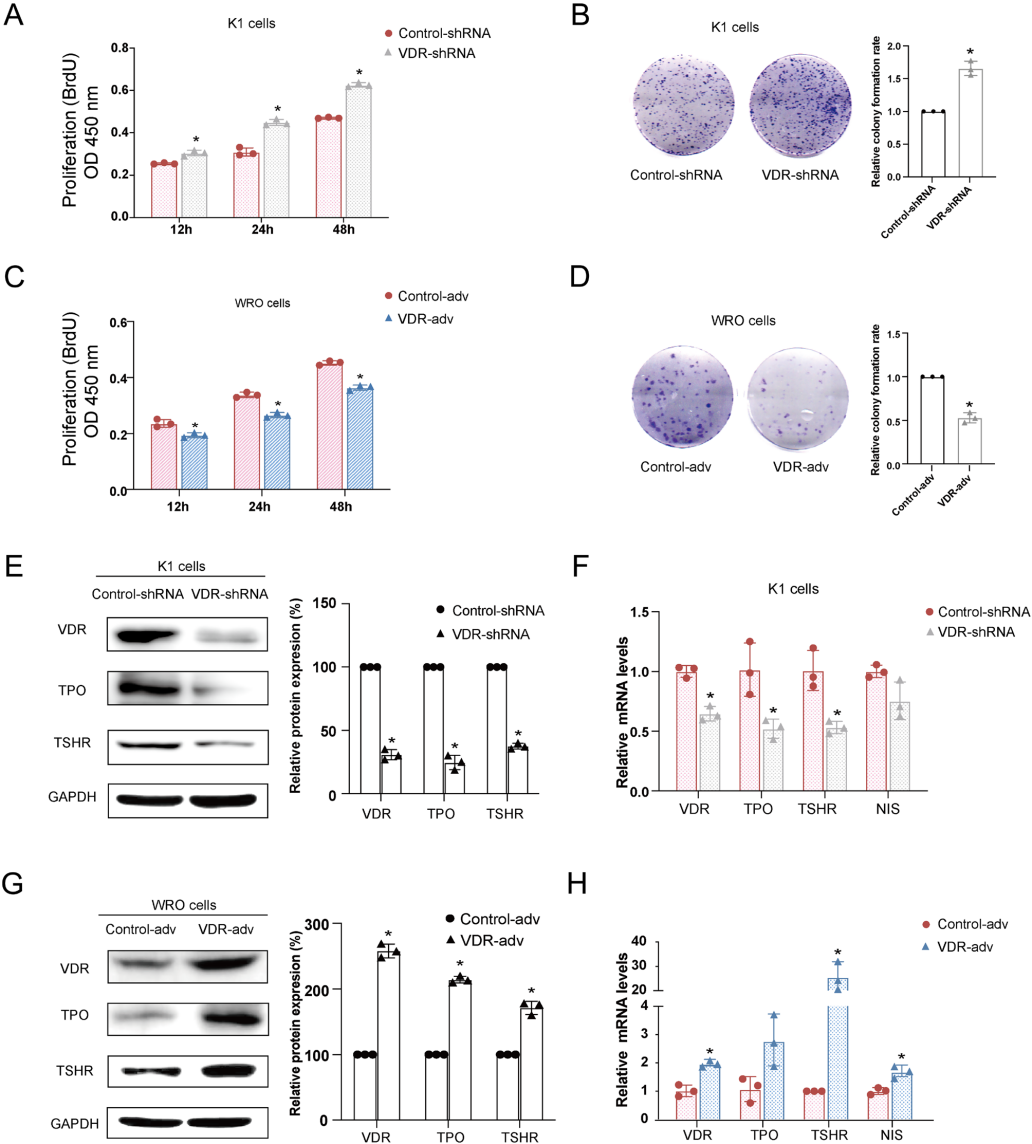
VDR suppressed the proliferation and promoted the differentiation of DTC cells* in vitro*. K1 cells were infected with lentivirus containing VDR shRNA (VDR-shRNA) or control shRNA (control-shRNA). Cell proliferation was determined by BrdU incorporation (A) and colony formation assay (B) after knockdown of VDR in K1 cells. WRO cells were infected with adenovirus containing VDR cDNA (VDR-adv) or control adenovirus (control-adv). Cell proliferation was determined by BrdU incorporation (C) and colony formation assay (D) after overexpression of VDR in WRO cells. (E–H) qRT-PCR and Western blotting analyses were performed to examine the effects of VDR overexpression or knockdown on differentiation markers including TPO, TSHR, NIS mRNA, and protein level. The data were presented as densitometric ratios normalized to GAPDH. Image J was used for WB quantification. Each assay was performed in triplicates (*n* = 3). Statistical values were analyzed by independent samples *t*-test or ANOVA analysis (*P  < 0.05 vs control). A full color version of this figure is available at https://doi.org/10.1530/JME-21-0167.

To further investigate the role of VDR in thyroid cancer tumorigenesis, we examined cell proliferation and differentiation of DTC cells in which VDR was underexpressed or overexpressed. Based on the protein expression level of VDR was higher in the K1 cell line than the WRO cell line (Fig. 1C), VDR in the K1 cell line was underexpressed by using VDR shRNA, and VDR in the WRO cell line was overexpressed by using the adenoviral system. The BrdU cell proliferation assay and colony formation assay were performed to assess the effects of VDR on the cell proliferation of DTC cells. Notably, knockdown of VDR increased the level of cellular proliferation and colony formation in K1 cells compared with the control group (Fig. 2A and  B), while overexpression of VDR inhibited levels of cell proliferation (Fig. 2C) and colony formation (Fig. 2D) in WRO cells. Moreover, knockdown of VDR reduced the expression of thyroid differentiation markers TPO and TSHR at two- to three-folds both in mRNA (Fig. 2F) and protein levels (Fig. 2E), while overexpression of VDR showed the opposite effects (Fig. 2G and  H). Taken together, these results suggest that VDR suppresses DTC cell proliferation and promotes DTC cell differentiation.

### Knockdown of VDR reduced E-cadherin expression in the membrane and increased β-catenin expression in the nucleus of DTC cells

**Figure 3:**
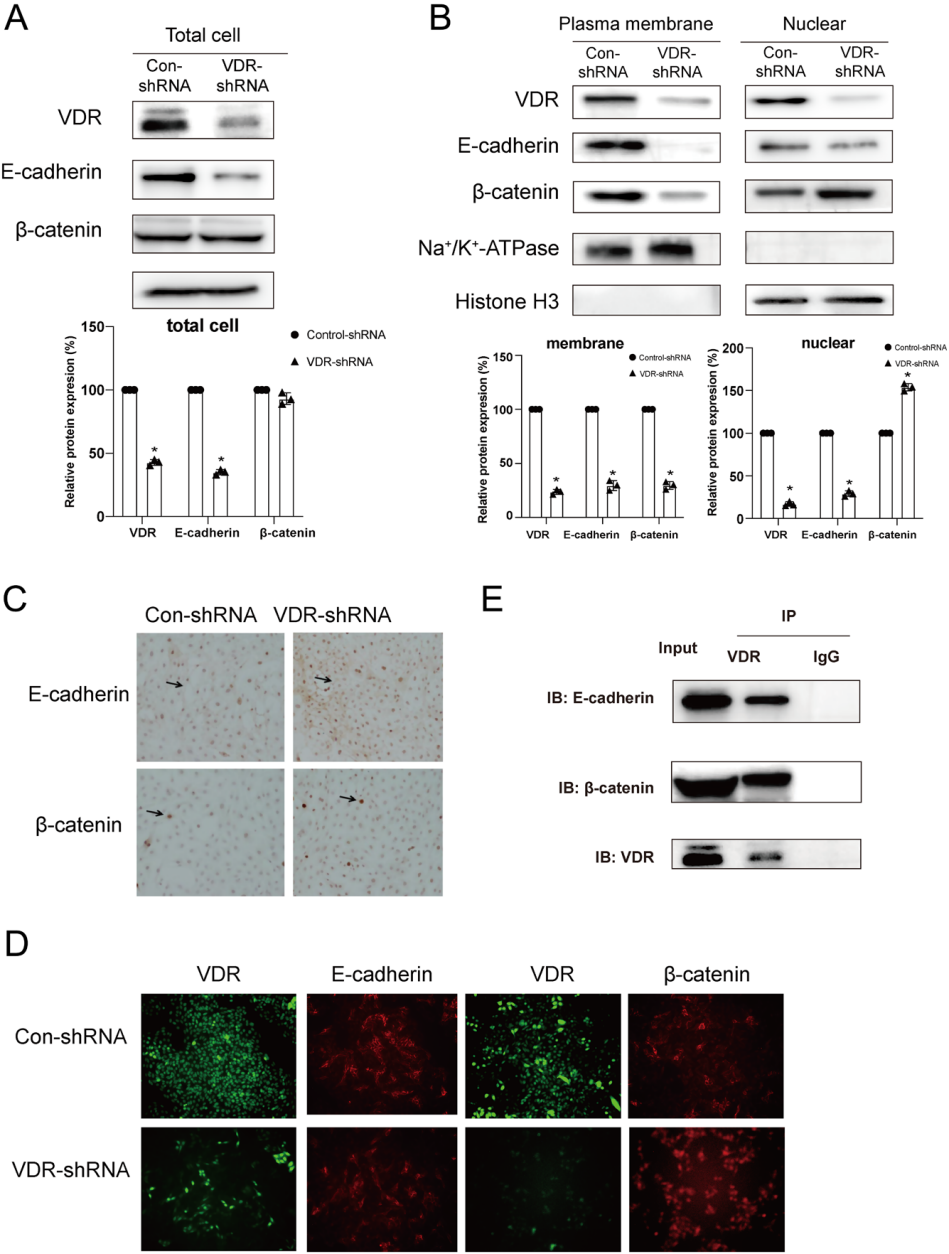
VDR increased E-cadherin expression in the membrane and reduced β-catenin expression in the nucleus of DTC cells. Cells were treated with VDR-shRNA and control-shRNA. (A) The E-cadherin and β-catenin total protein expression by Western blot. Densitometric quantification analysis results were presented in the below panel. The data were presented as densitometric ratios normalized to GAPDH. (B) Cell nuclear and membrane fractions were isolated. Western blot was performed to detect VDR, E-cadherin, and β-catenin. Na^+/^K^+^ATPase and Histone H_3_ were used as positive control for membrane and nuclear fractions, respectively. Densitometric quantification analysis results were presented in the below panel. The data were presented as densitometric ratios normalized to Na^+/^K^+^ATPase or Histone H_3_. (C) The immunocytochemistry of E-cadherin and β-catenin in cells with or without VDR knockdown. Positive staining of cells (brown) was indicated by arrows. (D) The immunofluorescence of VDR (green), E-cadherin, and β-catenin (red) in cells with or without VDR knockdown. (E) Co-IP experiments were performed for VDR using K1 cells membrane protein incubated with anti-VDR antibody or nonspeciﬁc IgG. After immunoprecipitation with anti-VDR antibody, Western blotting was performed with antibodies of VDR, E-cadherin and β-catenin, respectively. Nonspeciﬁc IgG was used as a negative control. Image J was used for WB quantification. Each assay was performed in triplicate (*n* = 3). Statistical values were analyzed by independent samples *t*-test or ANOVA analysis (*P  < 0.05 vs control). A full color version of this figure is available at https://doi.org/10.1530/JME-21-0167.

We examined the expression and location of E-cadherin and β-catenin in K1 cells with VDR knockdown. The results showed that knockdown of VDR reduced E-cadherin, but not β-catenin expression in K1 cells (Fig. 3A). To determine whether the localization of E-cadherin and β-catenin is regulated by VDR, we examined E-cadherin and β-catenin localization after VDR knockdown using cellular fractionation and Western blot analysis. Remarkably, the level of E-cadherin expression in the membrane was attenuated and the level of β-catenin expression in the nucleus was increased in K1 cells after VDR knockdown (Fig. 3B). As shown by immunocytochemistry and immunofluorescence, knockdown of VDR reduced the E-cadherin expression in the membrane and enhanced β-catenin expression in the nucleus (Fig. 3C and  D). This data suggest that VDR reduces E-cadherin expression and localization of β-catenin in the plasma membrane and enhances localization of β-catenin in the nucleus.

It is well known that E-cadherin and β-catenin form a complex on the cell membrane, where it inhibits the nuclear translocation of β-catenin. To confirm whether VDR interacts with E-cadherin and β-catenin in the K1 cell membrane, we isolated the plasma membrane protein of the K1 cells and performed co-immunoprecipitation (co-IP) assays. The results showed enriched coprecipitation of VDR with E-cadherin and β-catenin in the plasma membrane of K1 cells (Fig. 3E). These data indicate that VDR increases the levels of E-cadherin in the plasma membrane and decreases the levels of β-catenin in the nucleus and VDR binds to E-cadherin and β-catenin in the plasma membrane of the DTC cell.

### VDR inhibited proliferation and stimulated differentiation of DTC cells in an E-cadherin and β-catenin dependent manner

**Figure 4:**
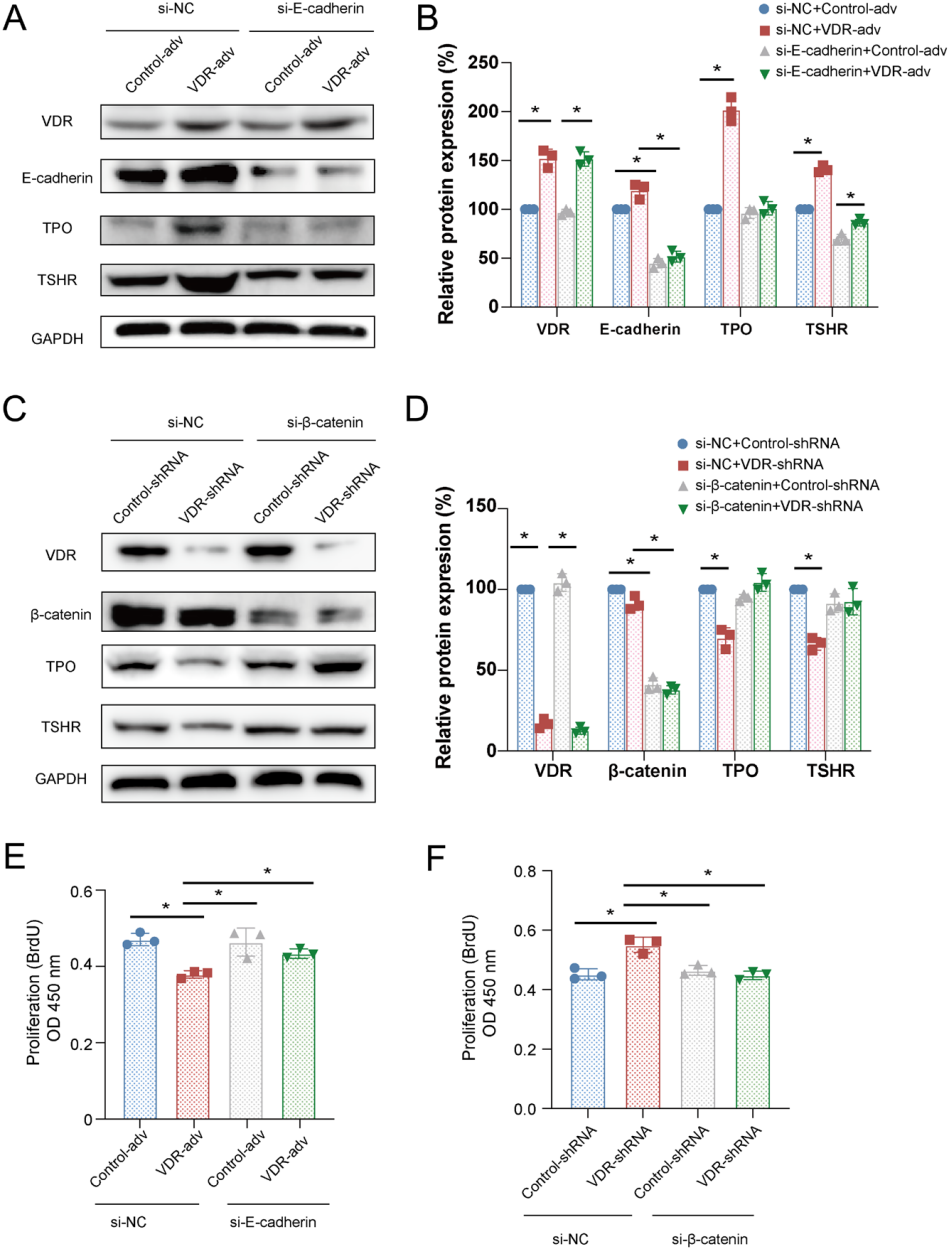
VDR reduced proliferation and increased differentiation of DTC cells in an E-cadherin and β-catenin dependent manner. WRO cells were treated with or without VDR-adv and then treated with control siRNA (si-NC) or E-cadherin siRNA (si-E-cadherin). K1 cells were transfected with or without VDR shRNA and then treated with control siRNA (si-NC) or β-catenin siRNA (si-β-catenin). (A, B, C, and D) Differentiation markers including TPO, TSHR, VDR, E-cadherin, and β-catenin were evaluated using Western blotting. The data were presented as densitometric ratios normalized to GAPDH. (E and F) Cell proliferation was determined by BrdU incorporation in DTC cells. Each assay was performed in triplicates (*n* = 3). Image J was used for WB quantification. Statistical values were analyzed by independent samples ANOVA analysis (*P  < 0.05 vs control). A full color version of this figure is available at https://doi.org/10.1530/JME-21-0167.

To determine the mechanism by which VDR regulates DTC cell proliferation and differentiation, we firstly investigated whether VDR regulates E-cadherin and β-catenin. The results showed that the expression of E-cadherin was decreased in DTC cells with VDR knockdown (Fig. 3A). To further delineate the role of E-cadherin in the proliferation and differentiation regulated by VDR, we use E-cadherin siRNA to knockdown the expression of E-cadherin in DTC cells in which VDR was overexpressed. As shown in Fig. 4, the knockdown of E-cadherin blocked VDR suppressed DTC cell proliferation (Fig. 4E) and induced TPO and TSHR expression as indicated by immunoblotting (Fig. 4A and  B). These data suggest that E-cadherin is required for VDR suppressed DTC cell proliferation and VDR-induced DTC cell differentiation.

Based on these observations, we hypothesized that β-catenin signaling mediates the inhibitory effects on DTC cell proliferation and motivating effects on DTC cell differentiation of VDR. To test the hypothesis, K1 cells were treated with VDR shRNA with or without β-catenin siRNA. As shown in Fig. 4C, D, and  F, knockdown of VDR resulted in increased DTC cell proliferation and decreased cell differentiation. However, the knockdown of β-catenin blocked the increased DTC cell proliferation and the suppressed DTC cell differentiation induced by VDR knockdown. These data suggest that β-catenin is required for VDR regulated proliferation and differentiation of DTC.

### VDR inhibited proliferation and stimulated differentiation in K1 cell-derived xenografts

To further verify the tumor-suppressive effect of VDR* in vitro* tumorigenesis assays, we examined the tumor formation in WRO or K1 cell xenografts. We found that only K1 cell xenografts developed visible tumors (data not shown). Then we investigated whether VDR suppresses tumor growth* in vivo* using a xenograft tumor model by subcutaneous injections of K1 cells into the flank of SCID mice (*n* = 6 for each group).

**Figure 5:**
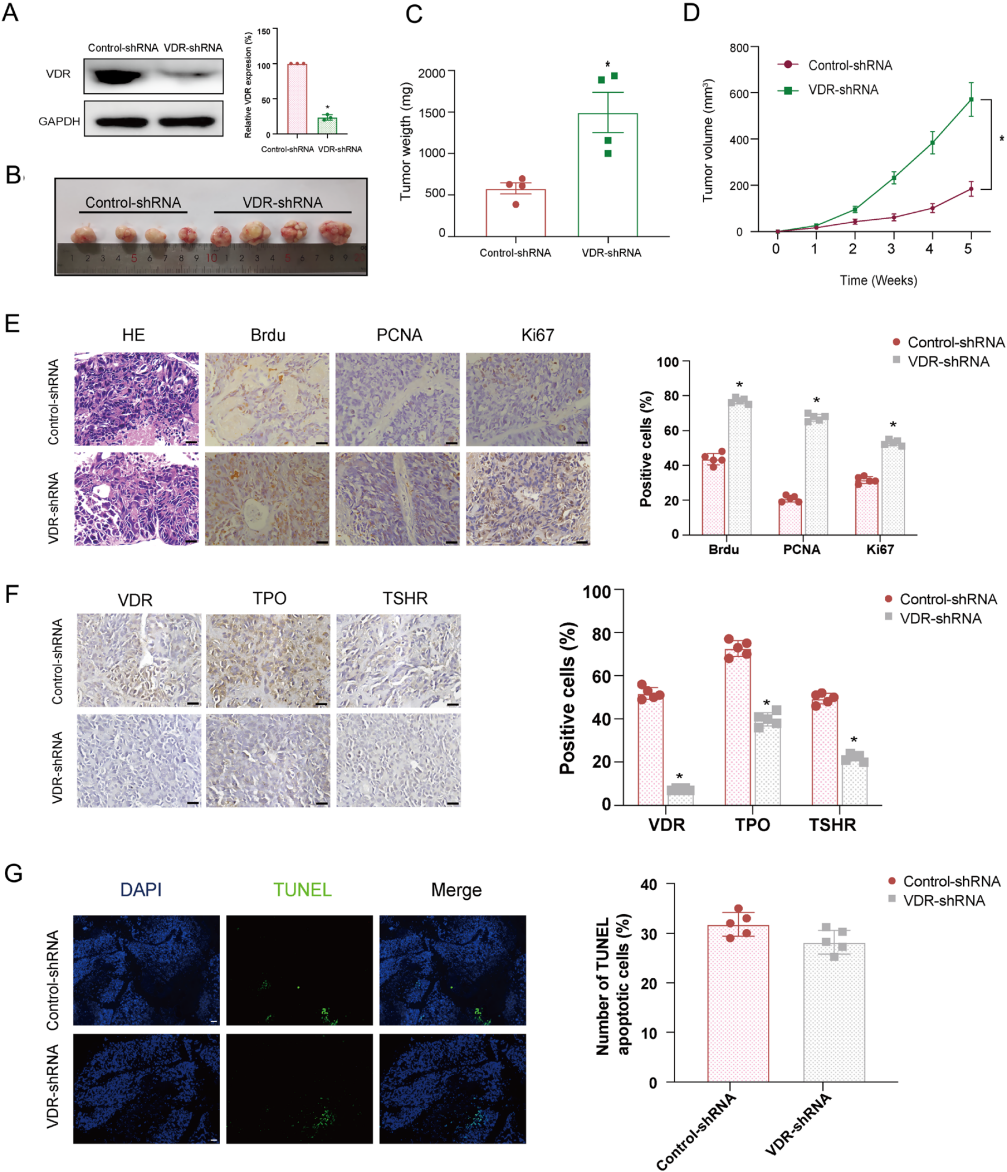
Effects of VDR knockdown on the proliferation and differentiation of DTC xenograft* in vivo*. The stable knockdown of VDR in K1 cells was established by selection with 2 μg/mL puromycin. K1 cells infected with control shRNA were used as a negative control. Then 5 × 10^6^ cells with VDR stable knockdown or control cells were implanted subcutaneously into the flanks of 8-week-old female SCID mice (*n*  = 6 for each group). Tumor diameters were measured once a week for five weeks. BrdU (50 mg/kg body weight) was injected intraperitoneally into mice 2 h before euthanization. (A) VDR level as detected by Western blotting in VDR stable knockdown and control K1 cells. (B) The representative views of isolated xenografts derived from VDR stable knockdown or control K1 cells. (C) The xenografts tumor weight of the VDR stable knockdown and control groups. (D) The tumor growth curve of the VDR stable knockdown and control groups. (E and F) The representative micrographs of H&E staining (left panel) and immunohistochemistry of BrdU, PCNA, Ki67, VDR, TPO, and TSHR in sections of xenografts with or without VDR knockdown. Positive staining of cells (brown) was indicated by arrows. The scare bar was 20 µm. (G) The representative views of TUNEL staining of xenografts with or without VDR knockdown. Apoptotic cells were stained with the green color. Nuclei were stained with DAPI (blue). Significance between two groups was analyzed by independent samples *t*-test and Mauchly’s test followed by sphericity assumed or modified Greenhouse–Geisser analysis was performed to distinguish within-subjects’ effects over time and between-subjects’ effects (*P  < 0.05 vs control). A full color version of this figure is available at https://doi.org/10.1530/JME-21-0167.

By using a retroviral system, VDR shRNA or control shRNA was stably expressed in K1 cells. As shown in Fig. 5A, the VDR shRNA decreased the expression of VDR by 80%. The results showed that tumor volume and weight were increased significantly in the VDR knockdown group when compared with the control group (Fig. 5B and  C).

The level of VDR was decreased in tumors with VDR knockdown than that in tumors with control shRNA (Fig. 5F). Moreover, VDR knockdown not only enhanced the expression of BrdU, PCNA, and Ki67 (Fig. 5E) but also reduced the expression of differentiation makers including TPO and TSHR (Fig. 5F). Meanwhile, the TUNEL assay showed no change in apoptosis of the xenografts with knockdown of VDR (Fig. 5G).

**Figure 6:**
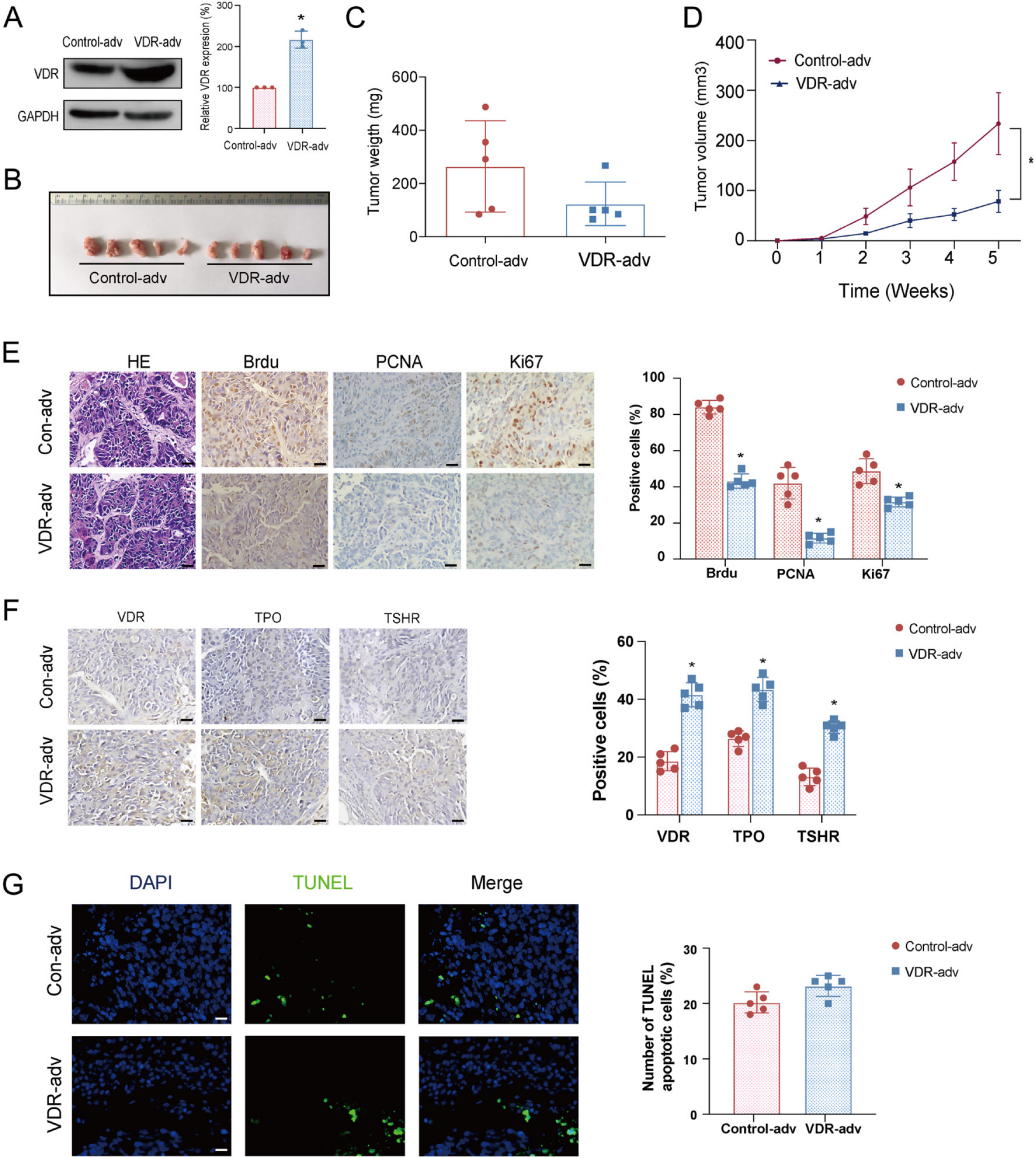
Effects of overexpression of VDR inhibits tumor formation of DTC xenograft in SCID mice. K1 cells (5 × 10^6^) with VDR stable overexpressed or control cells were subcutaneously injected into the flanks of 8-week-old female SCID mice (*n*  = 6 for each group). (A) VDR level as detected by Western blotting in VDR stable overexpression and control K1 cells. (B) The representative views of isolated xenografts derived from VDR stable overexpression or control K1 cells. (C) The tumor weight of the VDR stable overexpression and control groups. (D) The tumor growth curve of the VDR stable overexpression and control groups. (E and F) The macroscopic views of H&E staining (left panel) showing tumors upon resection from SCID mice. The immunohistochemistry of BrdU, PCNA, Ki67, VDR, TPO, and TSHR in sections of xenografts with or without VDR overexpression. Positive staining of cells (brown) was indicated by arrows. The scare bar was 20 µm. (G) the representative views of TUNEL staining of xenografts with or without VDR overexpression. The green color indicates apoptotic cells. Nuclei were stained with DAPI (blue). Significance between two groups was analyzed by independent samples *t*-test and Mauchly’s test followed by sphericity assumed or modified Greenhouse–Geisser analysis was performed to distinguish within-subjects’ effects over time and between-subjects’ effects (*P  < 0.05 vs control). A full color version of this figure is available at https://doi.org/10.1530/JME-21-0167.

To confirm the effect of VDR knockdown on DTC tumor growth, we examined the effects of overexpression of VDR in a xenograft experiment using SCID mice (*n*  = 6 for each group). The results showed that the expression of VDR in K1 cells was upregulated in about two folds as shown by immunoblotting (Fig. 6A). As shown in Fig. 6B, xenografts in the VDR overexpression group were smaller compared to the control group. The tumor volume and the mean tumor weight were lower in mice xenografted with VDR overexpressed K1 cells compared to those xenografted with cells transfected with the vector control (Fig. 6C and  D), indicating that VDR inhibits tumor growth. Consistent with the expression of VDR in K1 cells, the level of VDR was increased in the VDR overexpressed tumors than that in control tumors (Fig. 6F). The expression of BrdU, PCNA, and Ki67 were decreased in the VDR overexpressed tumors (Fig. 6E). However, TUNEL staining was not significantly different between the two groups (Fig. 6G), suggesting that VDR inhibits the DTC tumor growth and cell proliferation but does not affect apoptosis. Furthermore, the expression of TPO and TSHR in VDR overexpressed tumors was increased than those in control tumors (Fig. 6F), indicating that VDR promotes DTC tumor differentiation.

## Discussion

In the current study, we demonstrate that VDR was upregulated in DTC tissues than that in adjacent non-cancerous tissues, and the level of VDR positively correlates with differentiation of DTC. Furthermore, the results provide the first evidence that VDR suppresses proliferation and promotes differentiation both in DTC cells* in vitro* and DTC cell-derived xenografts* in vivo*. Besides, we demonstrate that VDR binds to the E-cadherin/β-catenin complex in the plasma membrane and works in an E-cadherin and β-catenin dependent manner. Taken as a whole, these results suggest that VDR is involved in DTC progression and will help uncover novel clues for the therapeutic strategy of DTC.

Our results confirm that VDR levels are highly expressed in DTC tissue than that in the adjacent non-cancerous tissue, which is consistent with the previous reports (Khadzkou *et al.* 2006, Zhang *et al.* 2018). Moreover, it has been reported that the expression of VDR is decreased in metastatic PTC and lost in the majority of ATC (Clinckspoor *et al.* 2012). Thus, the levels of VDR expression in malignant tissue of thyroid cancer are inversely associated with aggressive tumor characteristics, while increased expression of VDR in DTC might be one of the compensatory mechanisms for maintaining a relatively lower progression of the tumor. Several studies have reported that high VDR may be an important response against tumor progression (Wu *et al.* 2014). These results suggest that VDR has an important role in DTC progression. Therefore, we hypothesize that the increased expression of VDR in DTC tissues protects against DTC progression by suppressing the proliferation and maintaining the differentiation of DTC cells.

We explored the effect by which VDR was involved in PTC progression. In DTC cells, knockdown of VDR promoted cell proliferation as measured by BrdU assay and colony formation assay, and inhibited cell differentiation as demonstrated by suppressed expression of differentiation markers such as TSHR and TPO. In contrast, overexpression of VDR showed opposite effects. As lack of VDR ligands or the substrate for the synthesis of the ligand in the culture medium, the results suggest a role of VDR in inhibiting cell proliferation and promoting cell differentiation independent of its ligand. The ligand-independent role of VDR has also been demonstrated in the regulation of hair follicle cell differentiation (Hughes *et al.* 1988, Yoshizawa *et al.* 1997, Skorija *et al.* 2005). Our studies demonstrate for the first time the ligand-independent role of VDR in regulating proliferation and differentiation of DTC.

Consistent with our* in vitro* data, the results showed that VDR remarkedly delayed DTC tumor growth in xenograft in SCID mice. Furthermore, the knockdown or overexpression of VDR resulted in an increased or decreased proliferation and a decreased or an increased differentiation, respectively, indicating a tumor-suppressor role of VDR in DTC.

In the present studies, we investigated the molecular mechanism by which VDR inhibited DTC proliferation and promoted DTC differentiation. This study shows that overexpression of VDR increased the E-cadherin expression in DTC cell lines. However, when E-cadherin is reduced, the β-catenin complex is separated from the E-cadherin complex in the plasma membrane and accumulated in the cytoplasm. However, β-catenin would still stay in the cytoplasm because of binding with VDR in the cell plasma and not entering the nucleus. Therefore, the knockdown of E-cadherin had little effect on the nuclear translocation of β-catenin and cell proliferation and differentiation. Nevertheless, in DTC cell lines, inhibition of E-cadherin reversed the effects of VDR on the inhibition of cell proliferation and the stimulation of cell differentiation. These results suggest that E-cadherin mediates VDR-induced suppression of proliferation and stimulation of differentiation in DTC cells.

β-Catenin is mostly located in the nucleus of ATC cells. In contrast, β-catenin is mostly located in the plasma membrane and cytoplasm in DTC cells and rarely located in the nucleus. It has been reported that the level of β-catenin on the cell membrane significantly decreases while the level of β-catenin in the cytoplasm increases in poorly or undifferentiated thyroid cancer cells (Garcia-Rostan *et al.* 2001). This is probably why DTC cells had lower levels of proliferation and higher levels of differentiation compared to ATC cells. Therefore, our results showed that knockdown of β-catenin in DTC cells had little effects on cell proliferation and differentiation. Nevertheless, our data showed that knockdown of VDR increased levels of β-catenin in the nucleus and knockdown of β-catenin blocked the effects of VDR knockdown on proliferation and differentiation of DTC cells, suggesting that VDR regulates proliferation and differentiation of DTC cells via preventing β-catenin to enter the nucleus.

Importantly, VDR interacts with both E-cadherin and β-catenin in the plasma membrane as shown by the co-IP assay. Our present data showed that knockdown of VDR reduced E-cadherin expression in the membrane and increased β-catenin expression in the nucleus of DTC cells. Previous studies have shown that the E-cadherin/β-catenin complex plays a critical role in the proliferation and differentiation of multiple malignancies, including colon carcinoma (Pálmer *et al.* 2001), breast cancer (Rosso *et al.* 2014), prostate cancer (Zheng *et al.* 2017), and thyroid cancer (Cho *et al.* 2014). Given that β-catenin is mainly localized in the nucleus and VDR is lost in ATC (Garcia-Rostan *et al.* 1999, Clinckspoor *et al.* 2012), our data suggest the possibility that VDR regulates DTC cell proliferation and differentiation by stabilizing the E-cadherin/β-catenin complex in the membrane, therefore preventing β-catenin from entering the nucleus to stimulate T-cytokines/lymphocyte enhancers (TCF/LEF) transcriptional activity to activate the Wnt signaling pathway. Further studies are required to determine the molecule mechanism by which VDR interacts with E-cadherin and β-catenin and the relationship between VDR and β-catenin/Wnt signaling in DTC.

**Figure 7:**
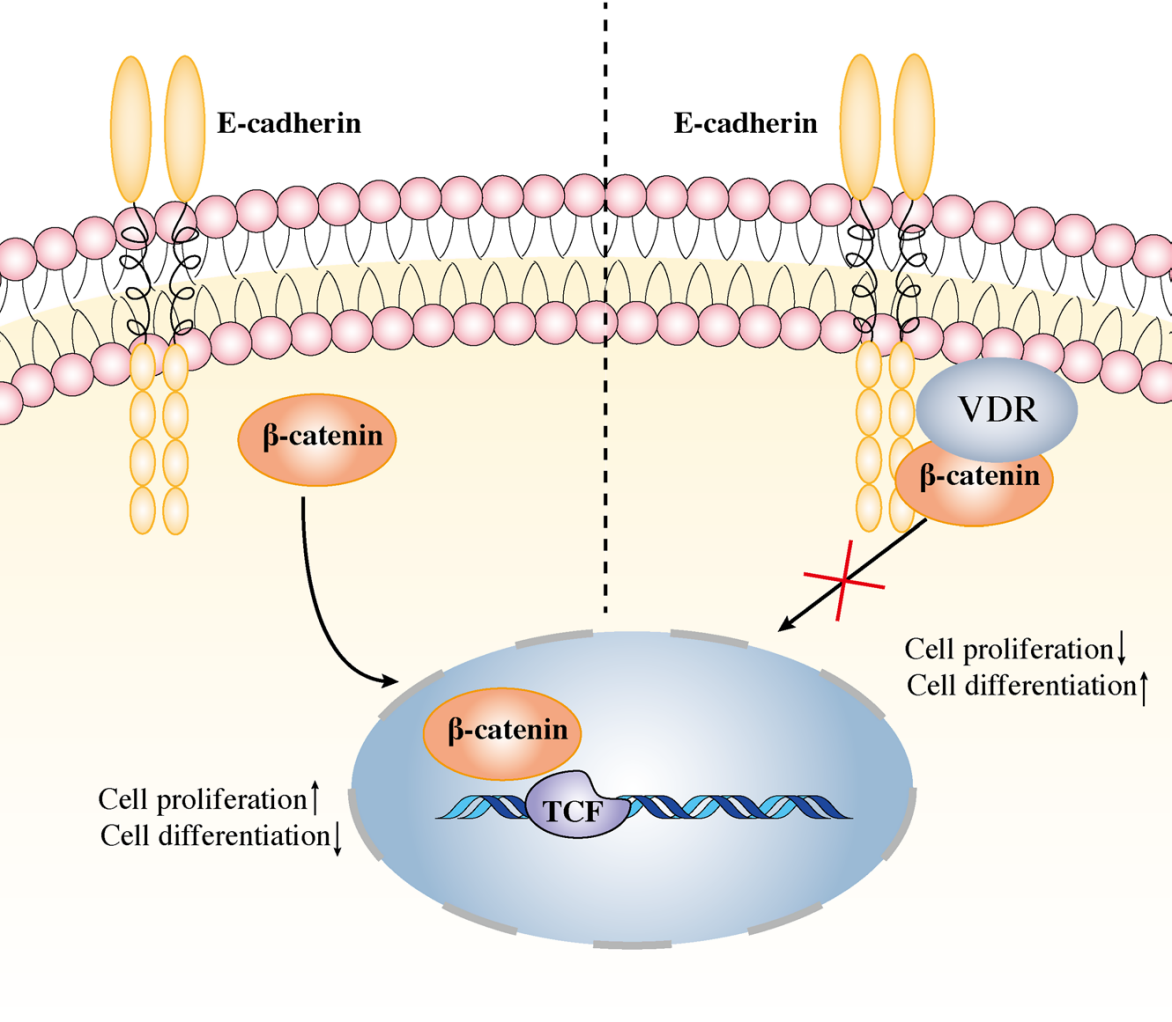
The potential mechanism by which VDR suppresses proliferation and promotes differentiation of DTC cells. When the VDR level is low in the cell membrane, the β-catenin dissociates from E-cadherin in the plasma membrane and enters the nucleus, which leads to cell proliferation. When the VDR level is normal or high, it binds to E-cadherin and β-catenin in the plasma membrane and subsequently prevents β-catenin from entering the nucleus, which inhibits cell proliferation and promotes cell differentiation. A full color version of this figure is available at https://doi.org/10.1530/JME-21-0167.

In conclusion, the present study points to the role of VDR in the inhibition of DTC cell proliferation and tumor growth and the stimulation of DTC cell differentiation via the E-cadherin and β-catenin signaling. Figure 7 shows the model which we proposed for the mechanism by which VDR inhibits DTC proliferation and promotes DTC differentiation.

## Conclusions

Taken together, our study revealed that VDR plays a key role in stabilizing the E-cadherin/β-catenin complex to inhibit proliferation and promote differentiation of DTC cells. This result not only provides new insights into the mechanisms explicating the upregulation of VDR in DTC but also suggests that the interaction of VDR with E-cadherin/β-catenin may provide a promising option for facilitating the research of thyroid cancer progression and will help uncover novel clues for the therapeutic strategy of DTC.

## Supplementary Material

SFigure 1. The expression level of VDR in the PTC and ATC tissues. Representative IHC staining for VDR in the PTC tissue and ATC tissue (n=3). ( positive staining cells staining brown, magnification, Left 100× and right 400×).

SFigure 2. VDR suppressed the proliferation and promoted the differentiation of DTC cells in vitro. (A-B) Fluorescence (blue excitation light source) under VDR downregulation and overexpression and control of WRO and K1 cells. (Green represents GFP fluorescence, virus-infected cells) (C-D) Cell proliferation was determined by BrdU incorporation after knockdown of VDR in WRO cells and overexpression of VDR in K1 cells. (E-H) Western blotting analyses were performed to examine the effects of VDR overexpression or knockdown on differentiation markers including TPO, TSHR protein level. Densitometric quantification analysis results were presented in the right panel. The data were presented as densitometric ratios normalized to GAPDH. Image J was used for WB quantification. Each assay was performed in triplicate (n=3). Statistical were analyzed by independent samples T-test or ANOVA analysis (*p < 0.05 vs control).

## Declaration of interest

The authors declare that there is no conflict of interest that could be perceived as prejudicing the impartiality of the research reported.

## Funding

This work was supported by the National Natural Science Foundation of China http://dx.doi.org/10.13039/501100001809 (grants 81672646 and 81471055).

## Data availability

All data generated or analyzed during this study are included in this published article or in the data repositories listed in References.

## Author contribution statement

Z X, Y L, and F X designed the study and wrote the manuscript. Y L, F X, X X, D D, and R S performed the experiment, collected the data, and conducted statistical analysis. All authors reviewed and approved the final version for submission.
