# Supplementary figures and images for: Vitamin D receptor regulates proliferation and differentiation of thyroid carcinoma via the E-cadherin-β-catenin complex
Source: J Mol Endocrinol. 2022 Jan 31;68(3):137–51. doi: 10.1530/JME-21-0167 (PMC8942331; doi:10.1530/JME-21-0167)

VDR (100×)

VDR (400×)

PTC

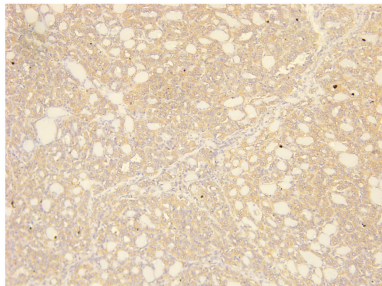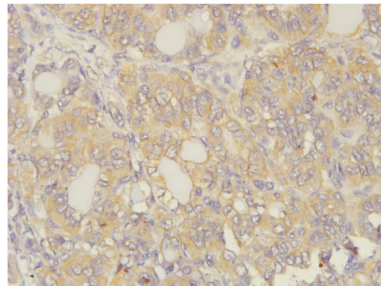

ATC

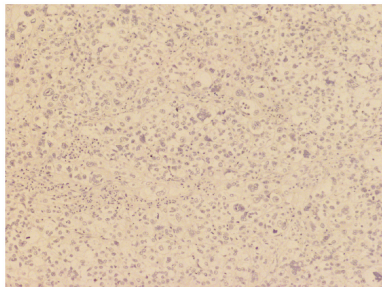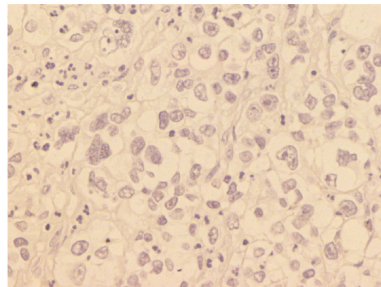

Supplement: SFigure 1. The expression level of VDR in the PTC and ATC tissues. Representative IHC staining for VDR in the PTC tissue and ATC tissue (n=3). ( positive staining cells staining brown, magnification, Left 100× and right 400×). [file supplementary_figure_1.pdf]

**A**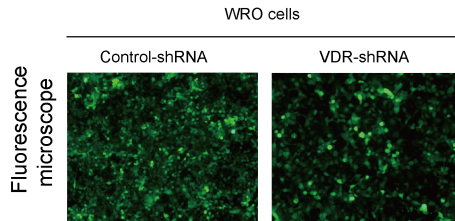**B**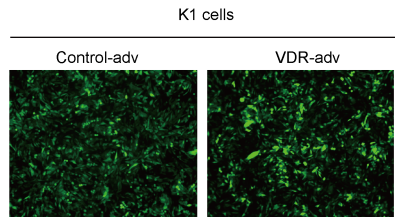**C**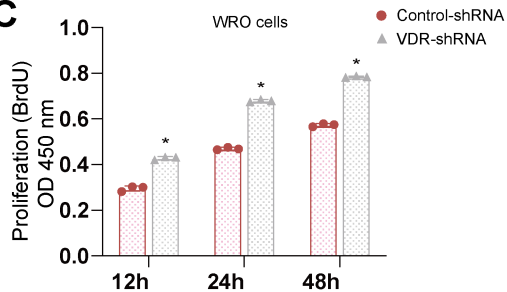**D**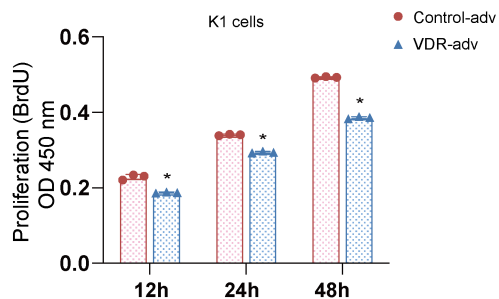**E**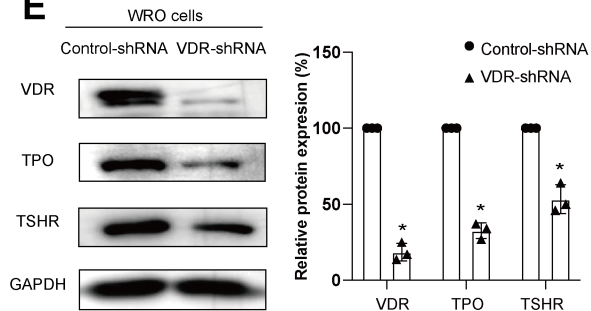**F**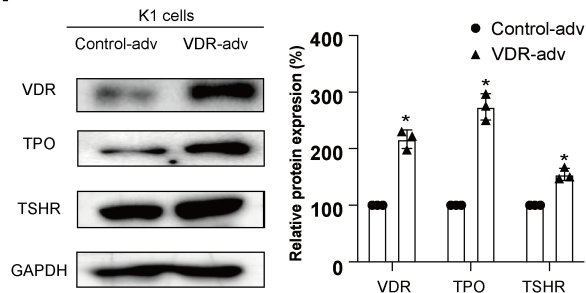

Supplement: SFigure 2. VDR suppressed the proliferation and promoted the differentiation of DTC cells in vitro. (A-B) Fluorescence (blue excitation light source) under VDR downregulation and overexpression and control of WRO and K1 cells. (Green represents GFP fluorescence, virus-infected cells) (C-D) Cell prol [file supplementary_figure_2.pdf]
